# A Graph Feature Auto-Encoder for the prediction of unobserved node features on biological networks

**DOI:** 10.1186/s12859-021-04447-3

**Published:** 2021-10-27

**Authors:** Ramin Hasibi, Tom Michoel

**Affiliations:** Computational Biology Unit, Department of Informatics, University of Bergen, Bergen, Norway

**Keywords:** Gene regulatory networks, Gene expression, Graph neural networks, Graph representation learning, Molecular networks, Omics, Feature prediction, Feature auto-encoder

## Abstract

**Background:**

Molecular interaction networks summarize complex biological processes as graphs, whose structure is informative of biological function at multiple scales. Simultaneously, omics technologies measure the variation or activity of genes, proteins, or metabolites across individuals or experimental conditions. Integrating the complementary viewpoints of biological networks and omics data is an important task in bioinformatics, but existing methods treat networks as discrete structures, which are intrinsically difficult to integrate with continuous node features or activity measures. Graph neural networks map graph nodes into a low-dimensional vector space representation, and can be trained to preserve both the local graph structure and the similarity between node features.

**Results:**

We studied the representation of transcriptional, protein–protein and genetic interaction networks in *E. coli* and mouse using graph neural networks. We found that such representations explain a large proportion of variation in gene expression data, and that using gene expression data as node features improves the reconstruction of the graph from the embedding. We further proposed a new end-to-end Graph Feature Auto-Encoder framework for the prediction of node features utilizing the structure of the gene networks, which is trained on the feature prediction task, and showed that it performs better at predicting unobserved node features than regular MultiLayer Perceptrons. When applied to the problem of imputing missing data in single-cell RNAseq data, the Graph Feature Auto-Encoder utilizing our new graph convolution layer called FeatGraphConv outperformed a state-of-the-art imputation method that does not use protein interaction information, showing the benefit of integrating biological networks and omics data with our proposed approach.

**Conclusion:**

Our proposed Graph Feature Auto-Encoder framework is a powerful approach for integrating and exploiting the close relation between molecular interaction networks and functional genomics data.

**Supplementary Information:**

The online version contains supplementary material available at 10.1186/s12859-021-04447-3.

## Introduction

Biological networks of genetic, transcriptional, protein–protein, or metabolic interactions summarize complex biological processes as graphs, whose structure or topology is informative of biological function at multiple scales. For instance, degree distributions reflect the relative importance of genes or proteins in a cell; 3–4 node network motifs have well-defined information-processing roles; and network clusters or communities contain genes or proteins involved in similar biological processes [1–3]. Simultaneously, genomics, transcriptomics, proteomics, and metabolomics technologies measure the variation or activity of genes, proteins, or metabolites across individuals or experimental conditions [4, 5]. There is a rich history of integrating the complementary viewpoints of biological networks and omics data. For instance, “active subnetwork” identification methods treat omics data as features of network nodes in order to identify well-connected subnetworks that are perturbed under different conditions [6]. Network propagation or smoothing methods on the other hand use biological networks to extend partial information on some nodes (e.g., disease association labels, partially observed data) to other nodes (e.g., to discover new disease-associated genes or impute missing data) [7, 8]. However, existing methods treat biological networks as discrete structures, which are intrinsically difficult to integrate with continuous node features or activity measures.

Recently, with the advent of deep learning, the idea of representation learning on graphs has been introduced. In this concept, nodes, subgraphs, or the entire graph are mapped into points in a low-dimensional vector space [9]. These frameworks are known as graph neural networks (GNNs), and use deep auto-encoders to preserve the local structure of the graph around each node in the embedding, without having to specify in advance what “local” means. However, not much attention has been paid so far to the representation of the node features in these embeddings [10, 11].

In this paper, we propose a new framework using graph representation learning on biological networks which results in embeddings that are compatible with or informative for molecular profile data, concentrating for simplicity on gene expression data. The three main contributions of this study are:

1. We introduce a method to systematically measure the relationship between the structure of a network and the node feature (gene expression) values. This is done using the Graph Auto-Encoder (GAE) approach of [12] and measuring (i) the performance of reconstructing the network from the embedding, with and without expression data, and (ii) measuring the variance in expression values explained by the embedding matrix.
2. We propose the framework of *Graph Feature Auto-Encoder (GFAE)* for the prediction of expression values utilizing gene network structures, and introduce a new convolution layer named FeatGraphConv using a message passing neural networks (MPNNs) framework, tailored to reconstructing the representation of the node features rather than the graph structure.
3. We show that our new approach to gene expression prediction has practical applications in tasks such as imputation of missing values in single cell RNA-seq data and similar scenarios.

## Related work on GNN

Assume that an undirected, unweighted graph ${\mathcal {G}}=({\mathcal {V}},{\mathcal {E}})$ with $N=|{\mathcal {V}}|$ number of nodes has an adjacency matrix **A**, where $A_{ij}=1$ if there is an edge between nodes *i* and *j* and zero otherwise, and degree matrix **D**, a diagonal matrix with the degrees (number of neighbours) of each node on the diagonal. Matrix ${\mathbf{X}} \in {\mathbb {R}}^{N\times Q}$, called the feature matrix, denotes node features. One of the first attempts at learning neural networks over graph structures was the convolution operation on graphs. For an input graph signal $x \in {\mathbb {R}}^N$, the spectral convolution is defined as

1 $$\begin{aligned} {\mathbf {g}}_\theta * x = {\mathbf {Ug}}_\theta {\mathbf {U}}^T x, \end{aligned}$$

in which ${\mathbf {U}}$ is the matrix of eigenvectors of the symmetric Laplacian ${\mathbf {L}}= {\mathbf {D}}-{\mathbf {A}}= {\mathbf {U}}{\varvec{\Lambda }}{\mathbf {U}}^T$. ${\mathbf {U}}^Tx$ is called the Fourier transform of signal *x* and ${\mathbf {g}}_\theta$ is a matrix function of $\varvec{\Lambda }$, the diagonal matrix of eigenvalues of ${\mathbf {L}}$.

Due to the high cost of calculating the eigenvalues in the case of large matrices, Hammond et al. [13] proposed to use a Chebyshev series expansion truncated after the $K^{th}$ term to approximate the graph convolution operation with a $K^{th}$-order polynomial:

2 $$\begin{aligned} {\mathbf {g}}_\theta * x \approx {\mathbf {U}}\sum _{k=0}^K \theta _k^{\prime} T_k (\tilde{\varvec{\Lambda }}) {\mathbf {U}}^T x=\sum _{k=0}^K\theta _k^{\prime} T_k(\tilde{\varvec{\Lambda }})x, \end{aligned}$$

in which $T_k(.)$ and $\theta _k^{\prime}$ are the $k^{th}$-order Chebyshev polynomials and expansion coefficients, respectively, $\tilde{\varvec{\Lambda }}=\frac{2}{\lambda _{max}}\varvec{\Lambda }- {\mathrm {I}}_N$ with $\lambda _{max}$ the largest eigenvalue of $\varvec{\Lambda }$ and ${\mathrm {I}}_N$ an identity matrix with size $N\times N$, and finally $\tilde{\mathbf {L}} = {\mathbf {U}}\tilde{\varvec{\Lambda }} {\mathbf {U}}^T = \frac{2}{\lambda _{max} }{\mathbf {L}}- {\mathrm {I}}_N$.

In the graph convolutional network (GCN) [14], further approximations were done by setting $K=1$, $\lambda _{max}\approx 2$, and $\theta = \theta _0^{\prime} = -\theta ^{\prime}_1$. As a result, formula (2) was transformed into

3 $$\begin{aligned} {\mathbf {g}}_\theta * x \approx ({\mathrm {I}}_N+{\mathbf {D}}^{-\frac{1}{2}}{\mathbf {AD}}^{-\frac{1}{2}})x. \end{aligned}$$

Repeated application of ${\mathbf {g}}_\theta$ resulting in high powers of ${\mathbf {D}}^{-\frac{1}{2}}{\mathbf {AD}}^{-\frac{1}{2}}$ can cause numerical instabilities. Kipf and Welling [14] suggested to set the diagonal elements of **A** to 1 (add self-loops) and to recompute **D** according to the updated adjacency matrix. Therefore, they used the symmetrically normalized adjacency matrix $\tilde{\mathbf{A }}$ in their convolution layer, with

4 $$\begin{aligned} \tilde{\mathbf {A}} = {\mathbf {D}}^{-1/2}{\mathbf {AD}}^{-1/2}. \end{aligned}$$

Thus, the forward operation in a GCN for ${\mathbf {X}}$ is computed as

5 $$\begin{aligned} {\mathrm {GCN}}({\mathbf {X}}, \tilde{\mathbf {A}})=\sigma (\tilde{\mathbf {A}}\,{\mathrm {ReLU}}(\tilde{\mathbf {A}}{\mathbf {XW}}_0){\mathbf {W}}_1) \end{aligned}$$

with weight matrices ${\mathbf{W} }_i$ containing the trainable weights for each input feature, and $\sigma$ a non-linear task specific function such as softmax for a node classification problem [10].

Additional studies on GNNs have shown that a GNN can be viewed as a message-passing approach based on graph structure, where every node’s message for its neighbours is the aggregation of the neighbourhood information, in which the aggregation is done through a trainable neural network [15]. This framework is also known as a MPNN. The forward pass in such a network consists of a message passing phase and a readout phase. In the message passing phase, the hidden representation of each node is updated through an update function, which aggregates the previous node representation and the messages of its neighbours according to:

6 $$\begin{aligned} h_{i}^k=\gamma ^k(h_{i}^{k-1},\,{\mathrm {Pool}}_{j\in N(i)}(M(h^{k-1}_i,h^{k-1}_j,e_{ij}))), \end{aligned}$$

in which $h_i^k$ is the hidden representation of node *i* in layer *k*, with $h^0_i$ being the node *i*’s input features and $e_{ij}$ is the edge attribute between nodes *i* and *j*. Additionally, $\gamma$ and *M* are both differentiable functions called the update and message functions, respectively and Pool is a permutation invariant pooling function. Furthermore, *N*(*i*) denotes the set of neighbouring nodes of node *i*.

In the readout phase, the feature vector of each node in the graph is computed using some learnable, differentiable readout function *R* according to

7 $$\begin{aligned} {\mathbf{Y}} = {\mathbf{R}} (\{h_i|i\in {\mathcal {V}}\}), \end{aligned}$$

in which, **Y** is the predicted labels. Different settings of $\gamma$, *M*, Pool, and **R** can lead to different MPNN convolution layers specific to different tasks and scenarios.

**Fig. 1:**
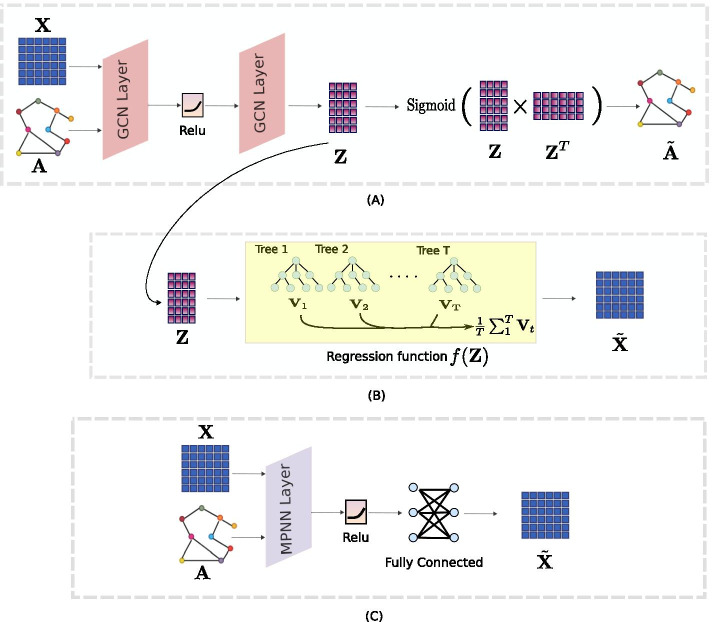
**A** Depicts the GAE scheme of [12] tailored to the reconstruction of the adjacency matrix of the graph. In **B** we take the embedding matrix of a GAE and train an indirect regression task for the prediction of the expression values. **C** Illustrates our proposed GFAE approach for end-to-end learning of graph node features

## Methods

In this section, we present two different GFAE frameworks of predicting expression values, leveraging the gene regulatory network structure, whose pipelines are depicted in Fig. 1. In this scenario, ${\mathbf{X}} \in {\mathbb {R}}^{N\times Q}$ is a matrix of expression values in *Q* different experiments for *N* number of genes (molecular profiles) and **A** denotes the adjacency matrix of the gene network.

### Structural embedding for indirect prediction of expression values

In this approach, we used the Non-probabilistic GAE model of [12] to represent the structure of a gene network as depicted in Fig. 1A. First the GCN operation, shown in formula (5), is modified by setting $\sigma$ in formula (5) to the identity function:

8 $$\begin{aligned} {\mathrm {GCN}}({\mathbf {X}},{\mathbf {A}}) = \tilde{\mathbf {A}}{\mathrm {ReLU}}(\tilde{\mathbf {A}}{\mathbf {XW}}_0){\mathbf {W}}_1, \end{aligned}$$

in which $W_0$ and ${\mathbf {W}}_1$ are trainable weights. Therefore, the embedding matrix of the graph adjacency ${\mathbf {Z}}$ is calculated by

9 $$\begin{aligned} { \mathbf {Z}}= GCN({\mathbf{X}} ,{\mathbf{A}} ). \end{aligned}$$

Furthermore, the weight matrices ${\mathbf {W}}_0$ and ${\mathbf {W}}_1$ in formula (8) are trained by measuring how well the embedding reconstructs the graph adjacency matrix, where the reconstructed adjacancy matrix $\hat{\mathbf{A }}$ is defined as

10 $$\begin{aligned} \hat{\mathbf{A }} = {\mathrm {Sigmoid}}({\mathbf{ZZ}} ^T). \end{aligned}$$

The cross-entropy error over all the edges in the matrix is used as a loss function,

11 $$\begin{aligned} {\mathcal {L}} = -\sum _{n=1}^{N} {\mathbf {A}}_{n}\,{\mathrm {ln}}\,\hat{\mathbf {A}}_{n}, \end{aligned}$$

where ${\mathbf {A}}_{n}$ and $\hat{\mathbf {A}}_n$ are the adjacency rows of the *n*th node in ${\mathbf {A}}$ and $\tilde{\mathbf {A}}$, respectively. The training of the neural network is done by gradient descent and stochasticity added by dropout rate. We use the metrics of average precision and area under the ROC curve related to the reconstruction of **A**, and the Variance Explained of ${\mathbf{X}}$ by **Z** to quantify the relationship between the node features and the graph structure.

As shown in Fig. 1B, the expression values of the genes are obtained following

12 $$\begin{aligned} \tilde{\mathbf{X }} = f({\mathbf{Z}} ) \end{aligned}$$

where $\tilde{\mathbf{X }}$ denotes the predicted expression values. Moreover, *f* is a regression function for which we consider linear regression (LR) and random forest (RF) regression as examples.

### Message passing neural network for end-to-end prediction of expressions

In the second method, we apply the message passing formula of (6) on the input expression values in which the messages (hidden representations) are propagated to each gene from neighbours in the gene network. As shown in Fig. 1C, in this approach, the model is trained in an end-to-end framework to predict the expression values directly from the input, without the need for training a separate regression model. To establish the performance of this framework, we used three popular message passing schemes for finding the hidden representation of the genes, as well as introducing our own, for the task of predicting gene expression values. These three methods are inductive GCN, GraphSAGE [16], and the GNN operator from [17] (from here on referred to as GraphConv). According to [15], a single GCN layer can also be viewed as a message passing scheme between the nodes in the graph in the format of formula (6):

13 $$\begin{aligned} h_i^k = \sum _{j\in N(i)\cup i}\frac{1}{\sqrt{deg(i)}*\sqrt{deg(j)}}.({\mathrm {W}}h_j^{k-1}), \end{aligned}$$

in which *deg*(*i*) is the number of neighbours of node *i*, W is a trainable weight matrix, and $\sum$ is the sum pooling operator. This scheme is equivalent to running a single GCN layer in formula (8). Another MPNN layer is GraphSAGE, whose formula is given by

14 $$\begin{aligned} h_i^k = {{\mathrm {W}}_1}(h^{k-1}_i) + {{\mathrm {W}}_2}{\mathrm {Mean}}_{j \in N(i)\cup i} (h^{k-1}_j), \end{aligned}$$

in which Mean is the mean pooling operator, and ${\mathbf {W}}_1$ and ${\mathbf {W}}_2$ are trainable weight matrices. GraphSAGE MPNN assumes that the representation of each node is the summation of the output from the previous layer and the average of the representation of the adjacent nodes. The final MPNN layer, named GraphConv, is calculated through

15 $$\begin{aligned} h_i^{k} = {\mathrm {W}}_1h_i^{k-1}+\sum _{j \in N(i)}{\mathrm {W}}_2.h_j^{k-1}, \end{aligned}$$

in which ${\mathbf {W}}_1$ and ${\mathbf {W}}_2$ are trainable weight matrices, and $\sum$ is the sum pooling operator. In this layer, the representation of each node is the sum product of the previous layer representation and the summation of the incoming messages from adjacent nodes.

In our proposed version of MPNN, FeatGraphConv, we first obtain a representation of every node’s features by running them through a linear layer in the message function *M*. This step helps the layer to find optimized message representations for the propagation phase. Then we aggregate the incoming neighbours’ messages by a mean pooling operator, based on the hypothesis that in a gene network, a gene’s expression value is intermediate between its neighbours expression values. For the update function $\gamma$, we concatenate the node’s embedding with its aggregated messages, and run them through a shared weight network, which determines how important each of these values are in predicting the features of the node. Hence, the formulae for our FeatGraphConv operator are as follows

16 $$\begin{aligned} \begin{aligned} g_i^{k}&= {\mathrm {W}}_{1} * h_i^{k-1}\\ h_i^{k}&= {\mathrm {W}}_{2}(g_i^{k}||{\mathrm {Mean}}_{j \in N(i)\cup i} (g^{k}_j)), \end{aligned} \end{aligned}$$

in which (.||.) is the concatenation function and ${\mathbf {W}}_1$ and ${\mathbf {W}}_2$ represent trainable weight matrices. In all of the four mentioned layers, the readout function **R** is defined as a fully connected linear layer. Thus, $\tilde{\mathbf{X }}_i$ the predicted expression values for node *i* are obtained through

17 $$\begin{aligned} \tilde{\mathbf{X }}_i = W * h_i + b, \end{aligned}$$

in which W and b represent trainable weights and bias matrices respectively. For finding the optimal weights in this framework, mean squared error (MSE) on predicted expression values is used as the loss function. This method is considered to be a semi-supervised training framework due to utilizing the complete structure of the graph in training the model.

### Prediction of expressions from expressions

For comparison of the results obtained through “Structural embedding for indirect prediction of expression values” and “Message passing neural network for end-to-end prediction of expressions” sections, we also consider prediction of $\tilde{\mathbf{X }}$ directly from **X** through simple machine learning algorithms. These algorithms include:

- *Multi layer perceptron (MLP)* A simple form of a neural network which maps the input features into output features through multiple layers of neurons (computing units).
- *Linear regression* A linear model for mapping the input to the output.
- *Random forest* A set of decision tree models that determine the output value through the aggregation of the output of decision trees that each are trained on a subset of **X**
- *Markov affinity-based graph imputation of cells (MAGIC)* Uses signal-processing principles similar to those used to clarify blurry and grainy images to recover missing values from already existing ones in a matrix [18]

## Experimental setup

In this section, we describe three experiments that are done to measure the relationship between gene network structure and expression values as well as thoroughly evaluate the performance of the proposed GFAE. The hyper-parameters of all the experiments were determined after some initial experiments on a separate validation set and were kept the same for all the models, to measure the predictive performance of different approaches under the same set of initial circumstances. These hyper-parameters are listed in Table 1. In order to make a sound and thorough performance evaluation, two masking methods are used to divide the data for training and evaluation, the details of which are explained below.

**Table 1:** Hyper-parameters of the graph neural network

Hyper-parameter	Node embedding	MPNN
Epochs	500	20,000
Initial learning rate	0.001	0.001
First hidden layer size	64	64
Second hidden layer size	32	32

**Fig. 2:**
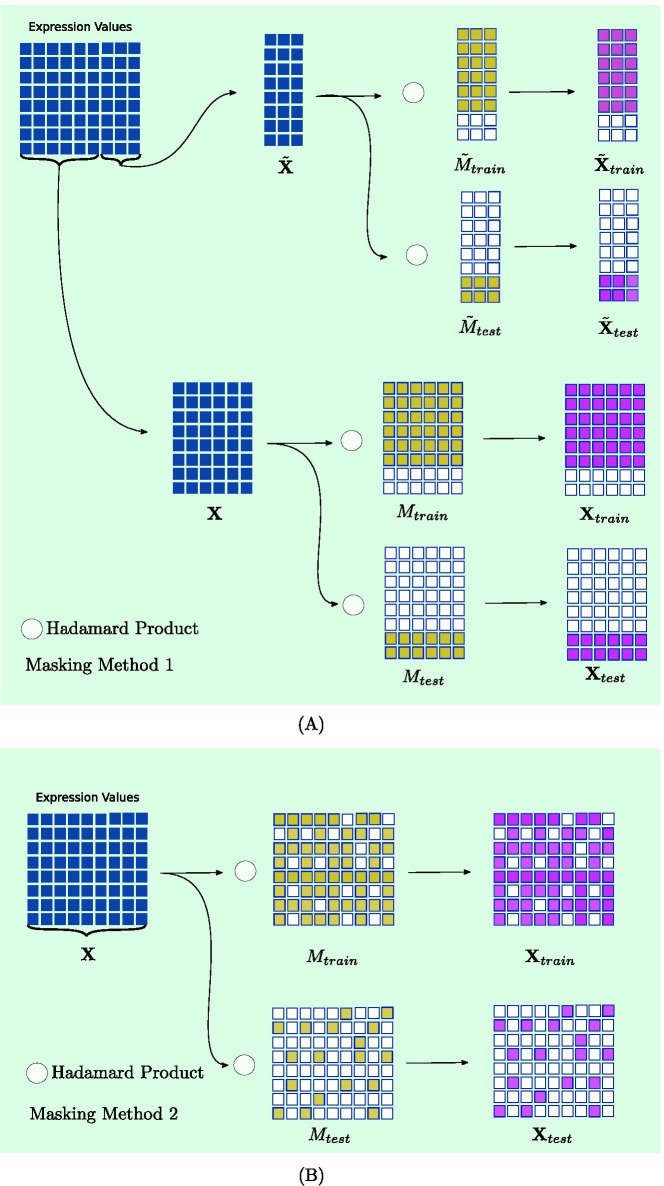
The two different approaches of masking of expression values. In **A**, each dimension of ${\mathbf {X}}_{test}$ is predicted using a separate model trained on ${\mathbf {X}}_{train}$. In **B**, a single model is used to predict all the values of the feature matrix

### Masking mechanism for the separation of train and test expression values

For evaluation purposes, we separate the expression values of ${\mathbf {X}}$ and $\tilde{\mathbf {X}}$ into two sets of training and testing. For this goal, two different masking techniques are used, the schemes of which are illustrated in Fig. 2. First, The input expression values ${\mathbf {X}}$ and the expression values that are to be predicted $\tilde{\mathbf {X}}$ are split into train and test through

18 $$\begin{aligned} {\mathbf{X}} _{train} &= M_{train}\circ {\mathbf{X}} , \qquad \tilde{\mathbf {X}}_{train} = \tilde{M}_{train}\circ \tilde{\mathbf {X}},\\ {\mathbf{X}} _{test} &= M_{test}\circ {\mathbf{X}} , \qquad \tilde{\mathbf {X}}_{test} = \tilde{M}_{test}\circ \tilde{\mathbf {X}}, \end{aligned}$$

where $\circ$ is the Hadamard product and $M_{train}, M_{test}, \tilde{M}_{train}, \tilde{M}_{test} \in \{0,1\}^{N\times Q}$ are binary matrices which have the value 1 in train and test indices, respectively. The goal is to train the models to predict the values of $\tilde{\mathbf {X}}_{train}$ using the values in ${\mathbf {X}}_{train}$ as input and evaluate the models when predicting $\tilde{\mathbf {X}}_{test}$ with ${\mathbf {X}}_{test}$ as input features. In the first masking method as depicted in Fig. 2A, the masking is done in such a way that both experiments (columns) and genes (rows) in expression profile matrix are split into separate train and test sets. Furthermore, in this approach, a model is trained to predict each column of the $\tilde{\mathbf {X}}$ independently (experiments based) to make the evaluation possible for regression functions, since they are only capable of predicting one value for each gene.

In the second masking mechanism (Fig. 2B), also refered to as the imputation masking, following the imputation mechanism in auto-encoders, we set $\tilde{\mathbf {X}}={\mathbf {X}}$ to measure the reconstruction ability of each model in an auto-encoder framework resulting in only two splits of ${\mathbf {X}}_{train}$ and ${\mathbf {X}}_{test}$. Thus, $M_{train}$ is set to 1 for some elements of ${\mathbf {X}}$ at random and $M_{test}$ is calculated as

19 $$\begin{aligned} M_{test} = \lnot M_{train}, \end{aligned}$$

where $\lnot$ indicates the logical not operator. Additionally, K-fold cross-validation is used in both masking techniques to ensure the soundness of all obtained results with K set to 10 or 3 depending on the time complexity of the specific experiment.

### Experiment on gene network structure embedding

In this experiment, we obtain the embedding matrix of the graph structure **Z** and measure the performance of the graph auto-encoder in calculating $\tilde{\mathbf {A}}$ (see “Structural embedding for indirect prediction of expression values” section). We used the PytorchGeometry implementation of the graph auto-encoder provided by [19]. For our approach, the normal auto-encoder provided in the package was used, and the variational auto-encoder was omitted.

Five different sets of input graphs and features to the model were tested:

1. *Random graph* In this approach ${\mathbf {Z}}$ is calculated by
  20 $$\begin{aligned} {\mathbf {Z}}= {\mathrm {GCN}}({\mathrm {I}}_N,{\mathbf {A}}_{rand}), \end{aligned}$$
  in which, ${\mathrm {I}}_N$ and ${\mathbf {A}}_{rand}$ represent an identity matrix of size $N \times N$ and the adjacency matrix of a random graph, respectively. For generating random graphs, we used the random graph generator of the Python3 package NetworkX, using the Erdős–Rényi model [20] (Additional file 1).
2. *Expression + random graph* In this approach, the identity matrix is replaced with the actual expression values of genes as input features:
  21 $$\begin{aligned} {\mathbf {Z}}= {\mathrm {GCN}}({\mathbf {X}},{\mathbf {A}}_{rand}). \end{aligned}$$
3. *Real graph* Following the approach used by [12], the embedding matrix ${\mathbf {Z}}$ in this case is calculated by
  22 $$\begin{aligned} {\mathbf {Z}}= {\mathrm {GCN}}({\mathrm {I}}_N,{\mathbf {A}}), \end{aligned}$$
  where **A** is the adjacency matrix of the input graph.
4. *Expression + real graph* The embedding **Z** in this case is calculated through formula (9),
  $$\begin{aligned} {\mathbf {Z}}= {\mathrm {GCN}}({\mathbf {X}},{\mathbf {A}}). \end{aligned}$$
5. *Expression* The network in this model, is inferred from the (absolute) correlation between the expression values of different genes. In this approach, the correlation directly outputs the probability of the edge between two nodes.

By choosing the identity matrix as input features in input setting 1 and 3, each of the nodes has a distinct set of features, which do not give any indication about the functionality of the node. This way the model will only pay attention to the graph structure when producing the embedding matrix. The edge set of the graph (${\mathcal {E}}$) is split into train and test sets and performance metrics of average precision (AP), area under ROC curve (AUC) on the test edge set, as well as the variance of ${\mathbf {X}}$ explained by ${\mathbf {Z}}$ are calculated for evaluation. The benefit of this experiment is that it allows to measure the relationship between the expression values and the structure of different gene networks through different metrics obtained from five different input settings as mentioned above.

### Experiment on the prediction of expression values using the proposed GFAE

In this experiment, the first masking mechanism (Fig. 2A and “Masking mechanism for the separation of train and test expression values” section) is used to evaluate different models for predicting expression values utilizing the structure of the network. Three sets of models are compared: indirect (Fig. 1B and “Structural embedding for indirect prediction of expression values” section), end-to-end framework (Fig. 1C and “Message passing neural network for end-to-end prediction of expressions” section), and baseline regression models (“Prediction of expressions from expressions” section). Moreover, two settings of inputs, $({\mathbf {X}}, A)$ and $({\mathrm {I}}_N,{\mathbf {A}})$, are used in the indirect and end-to-end models to compare their performance with and without input expression values. The purpose of this experiment is that it allows for a simple performance comparison between graph-based prediction methods in a sample regression task. Furthermore, prediction of expression values solely based on graph structure is possible in this setting. The average MSE for the prediction of each column of $\tilde{\mathbf {X}}_{test}$ is reported as the performance metric.

### Experiment on the imputation performance of the proposed GFAE

For this experiment, the second masking approach (“Masking mechanism for the separation of train and test expression values” section) or imputation masking is applied on the input ${\mathbf {X}}$ in end-to-end models (Fig. 1C and “Message passing neural network for end-to-end prediction of expressions” section). The goal of this experiment is to evaluate the proposed GFAE in imputation tasks such as the imputation of missing values in Single Cell RNA-seq datasets, as well as to compare the reconstruction ability of the proposed framework against traditional auto-encoders. The MSE of ${\mathbf {X}}_{test}$ is the metric used in this experiment to compare different auto-encoders.

**Table 2:** Summary description of benchmark datasets

		TF_net		PPI		Genetic	
Input	Expressions	Nodes	Edges	Nodes	Edges	Nodes	Edges
*E. coli*	466	1559	3184	1929	11,592	3688	147,475
Mus Musculus	1468	–	–	9951	75,587	–	–

### Datasets

We evaluated the performance of our method on data for the organisms *Escherichia Coli* (*E. coli*) and *Mus Musculus*.

- *Network datasets* For *E. coli*, we used transcriptional, protein–protein, and genetic interaction networks. The transcription network was obtained from RegulonDB [21]. All the positive and negative regulatory effects from the TF-gene interactions dataset file were included regardless of their degree of evidence (strong or weak) to construct the adjacency matrix. A PPI and genetic interaction network were obtained from BioGRID [22, 23]. We extracted the interactions from the file “BIOGRID-ORGANISM-Escherichia_coli_K12_W3110-3.5.180”, and considered the “physical” and “genetic” values of ’Experimental System Type’ column for constructing the PPI and genetic networks, respectively. For *Mus Musculus*, we used a protein–protein interaction network extracted in the same way from the file “BIOGRID-ORGANISM-Mus_musculus-3.5.182”.
- *Expression level dataset* For *E. coli*, we used the Many Microbes Microarray Database (M3DB) [24, 25]. All the experiments from the file “avg_E_coli_v4_Build_6_exps466probes4297” were used to construct the feature matrix. For *Mus Musculus*, the single cell RNA-Seq data from [26] were obtained from the Gene Expression Omnibus.

For each of the networks, the common genes between the network and the expression data were extracted, and an adjacency matrix and a matrix of features were constructed from the network and expression level datasets, respectively. A detailed description of each of the networks is available in Table 2.

### Computational resources and source code

All the experiments were done on a Tesla V100 with Python 3. The source code of the experiments is availabe at https://github.com/RaminHasibi/GraphFeatureAutoencoder.

**Fig. 3:**
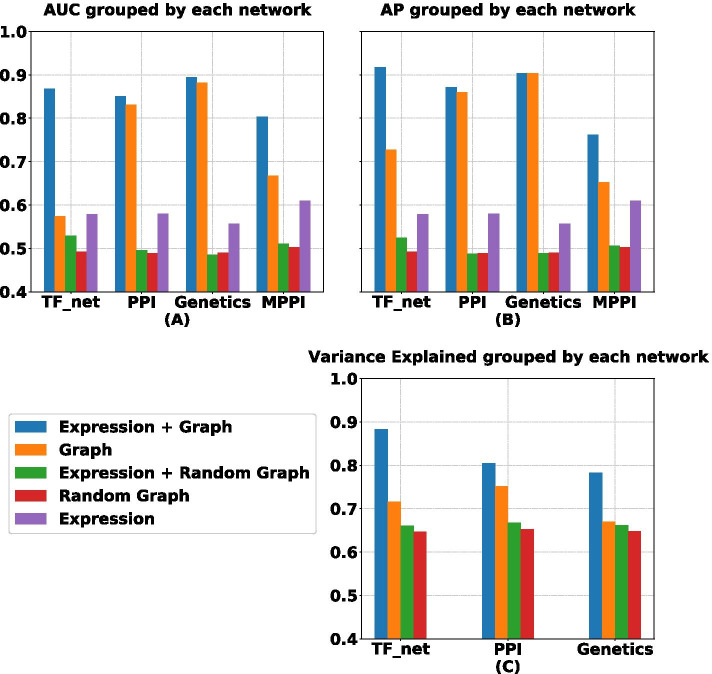
**A**, **B** Show AUC and AP of test set edge prediction on different gene networks given different input settings. **C** Shows the variance explained of the expression data matrix ${\mathbf {X}}$ by the embedding matrix **Z** (for exact values see the Additional file 1)

## Results

### Graph structural embeddings reconstruct gene networks and explain variation in gene expression

We obtained low-dimensional embeddings of transcriptional regulatory (TF_net), protein–protein interaction (PPI) and genetic interaction networks in *E. coli* and a PPI network in mouse (MPPI), with and without using expression data as node features, and trained the GAE to optimize the reconstruction of the original graph from the node embedding (see “Methods” section for details). Figure 3 shows the results for the graph reconstruction task for various embeddings. As seen in Fig. 3A, B, embeddings learned from the structure of the real graph alone (“Graph”) performed considerably better than embeddings learned from random graphs (“Random Graph”), as expected, in terms of both AUC and AP. The same was true for a standard Pearson correlation coexpression network inferred from the expression data alone (“Expression”), showing that graph embeddings and gene expression data independently predict graph structure.

When gene expression data were used as node feature inputs to the GAE (see “Structural embedding for indirect prediction of expression values” and “Experiment on gene network structure embedding” sections), graph reconstruction performance further increased (“Expression $+$ Graph” row), but this was not the case when expression data was combined with random graphs (“Expression $+$ Random Graph” row). In other words, graph embeddings where the distance between nodes respects both their graph topological distance and their expression similarity result in better graph reconstruction than embeddings that are based on topological information alone. This shows that expression profiles are informative of graph structure in a way that is consistent with, but different from, the traditional view where networks are inferred directly from expression data using expression similarity measures.

Next we computed the variance of the expression data explained by the different embeddings (see Fig. 3C and details in the Additional file 1). Despite being trained on the graph reconstruction task, graph embeddings learned with and without expression data as node features explained a high percentage of variation in the expression data, but not when random graphs were used.

In summary, graph representation learning results in low-dimensional node embeddings that faithfully reconstruct the original graph as well as explain a high percentage of variation in the expression data, suggesting that graph representation learning may aid the prediction of unobserved expression data.

### Indirect and end-to-end GFAE predict unobserved expression values

As mentioned in “Experiment on the prediction of expression values using the proposed GFAE” section, we considered three categories of prediction methods: (i) standard baseline methods that don’t use graph information (LR, RF, MLP, see “Prediction of expressions from expressions” section), (ii) standard regression methods trained on graph embeddings instead of directly on the training data (LR-embedding and RF-embedding, see “Structural embedding for indirect prediction of expression values” section), and (iii) graph MPNN methods for end-to-end learning of features (GCN, GraphSage, GraphConv, and FeatGraphConv, see “Message passing neural network for end-to-end prediction of expressions” section and Fig. 1. Table 3 shows the performance (average mean squared error) of all methods on the *E. coli* data. For this experiment the mouse single-cell RNA-seq data was omitted due to sparsity of the expression values. The “Features” and “Graph” columns indicate the input settings of $({\mathbf {X}}, A)$ and $({\mathrm {I}}_N,{\mathbf {A}})$, respectively.

The newly proposed graph convolution layer of FeatGraphConv is able to predict the unobserved expression values better than the other graph convolutions, due to the fact that this layer is tailored to the prediction of features rather than the reconstruction of the graph. As expected, all end-to-end methods perform considerably better when training data is included as node features. The end-to-end methods, with the exception of GCN, also perform better than the indirect methods where regression models are trained on graph embeddings. We also observe that the lowest MSE overall is in fact obtained by baseline LR on the training data alone. However, experiments on FeatGraphConv with 20,000 iterations (as opposed to the default of 500 used for all end-to-end methods in Table 3) showed that this model can decrease the MSE to $0.204\pm 0.12$, $0.133\pm 0.089$, and $0.107\pm 0.083$ for each of the TF_net, PPI, and Genetic networks, respectively, which is better than LR. However, due to the high number of experiments and the need to train a different model for each of the experiments of $\tilde{\mathbf {X}}_{test}$, it is not computationally efficient to train the more complex GNN models with a higher number of iterations by default for this prediction task.

On the other hand, when the graph structure alone is used (“Graph”), the indirect embedding-based methods achieve lower error. This could be due to the fact that these models better capture the structure of the graph, since their loss function is defined on the reconstruction of the adjacency matrix. Hence when the graph structure is the only information provided to the model, they are able to better capture this information and therefore obtain an embedding that better predicts expression data (on the basis of the results in “Graph structural embeddings reconstruct gene networks and explain variation in gene expression” section), compared to end-to-end models which try to predict the expression directly and are operating blindly when expression values are provided as input.

**Table 3:** The average MSE of predicting test expression values $\tilde{\mathbf {X}}_{test}$ using different models

Method	TF_Net		PPI		Genetics	
	Features	Graph	Features	Graph	Features	Graph
GCN	7.791 ± 3.550	15.127 ± 2.280	6.208 ± 0.607	11.106 ± 0.52198	5.988 ± 0.696	4.560 ± 0.351
GraphSAGE	0.332 ± 0.160	8.078 ± 2.592	0.265 ± 0.135	**2.844 ± 0.349**	0.233 ± 0.127	4.466 ± 1.605
GraphConv	0.318 ± 0.154	13.812 ± 4.534	0.308 ± 0.139	3.094 ± 0.431	0.234 ± 0.116	5.226 ± 1.054
FeatGraphConv	**0.285 ± 0.135**	**7.525 ± 2.941**	**0.244 ± 0.130**	5.207 ± 1.476	**0.201 ± 0.112**	**3.414 ± 0.691**
LR-embedding	**1.583 ± 0.200**	2.279 ± 0.403	1.091 ± 0.166	1.453 ± 0.264	**1.863 ± 0.332**	**1.653 ± 0.271**
RF-embedding	1.945 ± 0.318	**2.150 ± 0.363**	**1.472 ± 0.267**	**1.452 ± 0.267**	1.883 ± 0.343	1.897 ± 0.351
MLP	0.424 ± 0.170	–	0.354 ± 0.153	–	0.332 ± 0.134	–
LR	**0.215 ± 0.126**	–	**0.147 ± 0.105**	–	**0.108 ± 0.084**	–
RF	0.507 ± 0.143	–	0.194 ± 0.103	–	1.882 ± 0.343	–

(Bold indicates lowest error mean per category of experiments for each group of methods (end-to-end, indirect, or baseline)

### Graph feature auto-encoding improves the imputation of randomly missing values in single-cell RNA-seq data

Based on the results in the previous section, we next considered the more challenging prediction task where unobserved node features are randomly distributed over the nodes and differ between experiments, that is, the task of imputing randomly missing data (Fig. 2B). Since there are no fixed sets of training and test nodes, neither the baseline regression methods of LR and RF, nor the indirect frameworks are applicable in this case (“Structural embedding for indirect prediction of expression values” section). In contrast, the end-to-end GFAE methods allow to train a single model for the prediction of all the ${\mathbf{X}} _{test}$ values, which may be placed in any possible order inside the feature matrix. We used these models for the prediction of expression values in *E. coli* and of non-zero values in the single-cell RNAseq data in mouse, and benchmarked them against two methods that don’t use graph information, namely a normal MLP used in an auto-encoder scheme, and MAGIC, a method designed specifically to impute missing data in single-cell RNA-seq data [18] (see “Prediction of expressions from expressions” section).

As shown in Table 4, our FeatGraphConv convolution layer is able to predict missing features more accurately than all other methods. It is interesting to note that graph convolution layers, with the exception of GCN, outperform MAGIC on the single-cell RNAseq imputation task, although the MLP, which does not use graph information, also performs well in this case. The under-performance of GCN in these experiments can be explained by the fact that a GCN is primarily concerned with the structure representation of each node through multiplication of the degrees of neighbouring nodes (formula (13)). This captures the graph structure well, but has a negative effect on the prediction of node features. This is evident from the fact that other convolution layers that did not take node degrees into consideration performed better in the tasks given.

**Table 4:** The average MSE of predicting randomly distributed test values using different auto-encoder models

Model	*E. coli*			*Mus Musclus*
	TF_Net	PPI	Genetics	PPI
GCN	0.043 ± 0.00175	0.065 ± 0.004	0.114 ± 0.004	0.011 ± 0.001
GraphSAGE	0.027 ± 0.0007	0.023 ± 0.0004	0.026 ± 0.0003	0.004 ± 0.0006
GraphConv	0.041 ± 0.003	0.068 ± 0.05	0.182 ± 0.046	2.06 ± 2.73
FeatGraphConv (our)	**0.025 ± 0.0008**	**0.023 ± 0.0006**	**0.025 ± 0.0004**	**0.003 ± 0.0002**
MLP Auto-encoder	0.031 ± 0.0007	0.028 ± 0.0003	0.027 ± 0.0004	0.004 ± 0.0005
MAGIC	3.505 ± 0.006	3.661 ± 0.017	3.215 ± 0.003	0.050 ± 0.0002

(Bold indicates lowest error mean per network)

## Discussion

In this paper we studied whether GNN, which learn embeddings of nodes of a graph in a low-dimensional space, can be used to integrate discrete structures such as biological interaction networks with information on the activity of genes or proteins in certain experimental conditions. Traditionally, this is achieved by for instance network propagation methods, but these methods do not extract quantitative information from a graph that could be used for downstream modelling or prediction tasks. GNN on the other hand can include node features (gene or protein expression levels) in the learning process, and thus in theory can learn a representation that better respects the information contained in both data types. Thus far the integration of node features in graph representation learning has mainly been pursued for the task of link prediction. Here instead we focused on the task of predicting unobserved or missing node feature values.

We showed that representations learned from a graph and a set of expression profiles simultaneously result in better reconstruction of the original graph and higher expression variance explained than using either data type alone, even when the representations are trained on the graph reconstruction task. We further proposed a new end-to-end GFAE which is trained on the feature reconstruction task, and showed that it performs better at predicting unobserved node features than auto-encoders that are trained on the graph reconstruction task before learning to predict node features.

Predicting or imputing unobserved node features is a common task in bioinformatics. In this paper we demonstrated the value of our proposed GFAE on the problem of imputing missing data in single-cell RNAseq data, where it performs better than a state-of-the-art method that does not include protein interaction data. Other potential application areas are the prediction of new disease-associated genes from a seed list of known disease genes on the basis of network proximity [7], or the prediction of non-measured transcripts or proteins from new low-cost, high-throughput transcriptomics and proteomics technologies that only measure a select panel of genes or proteins [27, 28] which we intend to look into in our future works.

A potential drawback of our method is that it assumes that the interaction graph is known and of high-quality. Future work could investigate whether it is feasible to learn graph representations that can do link prediction and node feature prediction simultaneously, or whether network inference followed by graph representation learning for one type of omics data (e.g. bulk RNAseq data) can aid the prediction of another type of omics data (e.g. single-cell RNAseq).

In summary, our GFAE framework is a stepping stone in a new direction of applying graph representation learning to the problem of integrating and exploiting the close relation between molecular interaction networks and functional genomics data, not only for network link prediction, but also for the prediction of unobserved functional data.

## Supplementary Information

**Additional file 1.** Appendix of the paper containing extra information regarding the experiments.

## Data Availability

The source code of this project is written in python and is available on https://github.com/RaminHasibi/GraphFeatureAutoencoder.

## Abbreviations

GNN: Graph neural networks
LR: Linear regression
RF: Random forest
GCN: Graph convolution networks
MPNN: Message passing neural network
MLP: Multi layered perceptron
MAGIC: Markov affinity-based graph imputation of cells
MSE: Mean square error
TF_net: Transcriptional regulatory network
PPI: Protein–protein interaction
AUC: Area under the ROC curve
AP: Average precision
