# Supplementary material for: A Graph Feature Auto-Encoder for the prediction of unobserved node features on biological networks
Source: BMC Bioinformatics. 2021 Oct 27;22:525. doi: 10.1186/s12859-021-04447-3 (PMC8554915; doi:10.1186/s12859-021-04447-3)
Supplement: Supplementary file 1 — Additional file 1. Appendix of the paper containing extra information regarding the experiments. [file 12859_2021_4447_MOESM1_ESM.pdf]

A Graph Feature Auto-Encoder for the Prediction of Unobserved Node Features on Biological Networks

Hasibi, Ramin
Ramin.Hasibi@uib.no

Michoel, Tom
tom.michoel@uib.no

Computational Biology Unit, Department of Informatics

University of Bergen,

Norway

Appendix

A.1 Variance explained on features

For an embedding matrix $\mathbf{Z} \in \mathbb{R}^{N \times H}$ and feature matrix $\mathbf{X} \in \mathbb{R}^{N \times M}$, we calculated the amount of variance in $\mathbf{X}$ explained by $\mathbf{Z}$ as

$$\mathbb{V}_{\mathbf{Z}} = \frac{\mathrm{tr}\big(\mathbf{P_Z X}^T \mathbf{X}\big)}{\mathrm{tr}(\mathbf{X}^T \mathbf{X})} \tag{A1}$$

where $\mathbf{P_Z}$ is the projection matrix onto the subspace of $\mathbb{R}^N$ spanned by the columns of $\mathbf{Z}$,

$$\mathbf{P_Z} = \mathbf{Z}(\mathbf{Z}^T\mathbf{Z})^{-1}\mathbf{Z}^T.$$

Note that if we write the eigendecomposition of $\mathbf{X}^T\mathbf{X}$ as $\mathbf{X}^T\mathbf{X} = \mathbf{V}^T\boldsymbol{\Delta}\mathbf{V}$, then the columns of $\mathbf{V}$ corresponding to the nonzero eigenvalues in $\boldsymbol{\Delta}$ are the principal components of $\mathbf{X}$. If $\mathbf{Z}$ consist of the ith principal component, then eq. (A1) reduces to the familiar variance explained by this component, $\Delta_i/(\sum_j \Delta_j)$. If $\mathbf{Z}$ consists of a single vector $z \in \mathbb{R}^N$ with unit length, $\|z\| = 1$, then eq. (A1) reduces to

$$\mathbb{V}_z = \sum_{i=1}^{N} \frac{\Delta_i}{\sum_j \Delta_j} (z^T u_i)^2,$$

a weighted sum of the variances explained by each principal component, with weights determined by the extent of overlap between z and each principal component. Eq. (A1) generalizes this to summing the variances explained by multiple vectors simultaneously that need not be mutually orthogonal.

A.2 Random Graph Generation using Erdő s–Rényi model

According to Erdő s–Rényi, a random graph $G(n, p)$ has $\binom{n}{2}p$ edges placed at random. The degree distribution of each node is calculated through:

$$P(\deg(v) = k) = \binom{n-1}{k} p^k (1-p)^{n-1-k}, \tag{A2}$$

which is the binomial distribution. We adjusted the value of p so that the random graphs would approximately have the same number of edges as the real networks.

A.3 Results of gene regulatory networks reconstruction and gene expression variance explained

Input	AUC				AP			
	Ecoli			Mus Musculus	Ecoli			Mus Musculus
	TF_net	PPI	Genetics	PPI	TF_net	PPI	Genetics	PPI
Expression + Graph	0.868±0.017	0.8502±0.002	0.894±0.01	0.803±0.000	0.918±0.007	0.872±0.003	0.904±0.006	0.762±0.000
Graph	0.574±0.020	0.8316±0.018	0.882±0.014	0.6674±0.0000	0.727±0.012	0.860±0.010	0.904±0.006	0.653±0.000
Expression + Random Graph	0.529±0.01	0.4958±0.002	0.485±0.005	0.5108±0.0000	0.525±0.011	0.488±0.001	0.489±0.004	0.506±0.000
Random Graph	0.492±0.006	0.4892±0.006	0.490±0.007	0.5025±0.0000	0.496±0.005	0.486±0.005	0.5002±0.003	0.505±0.000
Expression	0.579±0.02	0.580±0.018	0.557±0.02	0.610±0.000	0.611±0.002	0.624±0.018	0.590±0.02	0.627±0.002

Table A1: Area under the ROC curve (AUC) and average precision (AP) for reconstructing biological network in E. coli and mouse from five graph structure embedding approaches

Table A2: The average Variance Explained on Gene Expression from the Regulatory Networks graph embedding

Input	Ecoli		
	TF_net	PPI	Genetics
Expression + Graph	0.883±0.005	0.805±0.003	0.7824±0.006
Graph	0.716±0.010	0.752±0.008	0.6697±0.010
Expression + Random Graph	0.660±0.010	0.667±0.013	0.6618±0.014
Random Graph	0.647±0.017	0.652±0.018	0.6483±0.010
